# An exploration of information exchange by adolescents and parents participating in adolescent idiopathic scoliosis online support groups

**DOI:** 10.1186/s13013-016-0084-9

**Published:** 2016-08-11

**Authors:** Traci Schwieger, Shelly Campo, Keli R. Steuber, Stuart L. Weinstein, Sato Ashida

**Affiliations:** 1 Department of Biostatistics, The University of Iowa, 2400 University Capitol Centre, Iowa City, Iowa 52242 USA; 2 Department of Community and Behavioral Health, The University of Iowa, Iowa City, Iowa 52242 USA; 3 Department of Communication Studies, The College of New Jersey, Ewing, New Jersey 08628 USA; 4 Department of Orthopaedics and Rehabilitation, The University of Iowa, Iowa City, Iowa 52242 USA

**Keywords:** Scoliosis, Brace, Spine, Deformity, Internet, Support groups, Adolescent idiopathic scoliosis, Online

## Abstract

**Background:**

Research indicates that healthcare providers frequently fail to adequately address patients’ health information needs. Therefore, it is not surprising that patients or parents of a sick child are seeking health information on the internet, in particular in online support groups (OSGs). In order to improve our understanding of the unmet health information needs of families dealing with adolescent idiopathic scoliosis (AIS), this study assessed and compared the types of information that adolescents and parents are seeking in OSGs.

**Methods:**

This study used two publicly accessible AIS-related OSGs on the National Scoliosis Foundation (NSF) website that targeted those who are receiving brace treatment and those under observation without treatment. Information exchanges were coded as providing or seeking information. Types of information being exchanged were categorized into several AIS-specific and brace-specific categories. Through a review of over 8,000 messages, 305 adolescents with AIS and 300 parents of a child with AIS were identified and categorized based on stage of illness/treatment. One message from each individual was randomly selected and coded for analysis.

**Results:**

There were significantly more (*p* < 0.001) parents that had a recently diagnosed child compared to recently diagnosed adolescents participating in the AIS-related OSGs, whereas there were significantly more (*p* = 0.004) adolescents that experienced brace treatment compared to parents of a child that experienced brace treatment. The most frequent information exchanged by adolescents and parents was AIS-related concerns regarding causes, diagnosis, and progression of the condition. However, compared to adolescents, parents exchanged this AIS-related information significantly more (*p* < .001) in their posts. Finally, compared to parents, adolescents exchanged significantly more information about appearance-related concerns regarding both AIS-related deformity (*p* < 0.002) and wearing a brace (*p* < 0.001).

**Conclusion:**

Families dealing with AIS are participating in OSGs to exchange information, in particular information related to the condition and to treatment. This study found similarities and differences regarding how information was exchanged (providing or seeking) and regarding frequency and types of information exchanged. Knowledge of these similarities and differences may be useful for improving health communication in the healthcare setting, at home, and for development and improvement of AIS-related website support.

## Background

Failure to fully address patients’ and families’ health information needs in the healthcare setting has frequently been found in the literature regarding a variety of illnesses [1–5]. Therefore, many patients are seeking additional health information outside of the hospital setting, in particular on the internet. For many, participation in online support groups (OSGs) can be a source of illness-related information and social support that may help to provide a sense of empowerment through increased acceptance, confidence, optimism, and enhanced social well-being [6–9]. Ultimately, the information and support provided by OSGs may impact several aspects of the patient’s illness including, coping with the illness, medical decision-making, behavior changes, preventative behaviors, and expectations.

Research indicates that parents of a child with chronic illnesses are seeking health information in OSGs [10, 11]. Unfortunately, little is known about the use of the internet to obtain health information for adolescents with chronic illnesses and whether the use of the internet as a source for health information differs between adolescents and their parents. One such chronic condition impacting both parents and adolescents is adolescent idiopathic scoliosis (AIS).

Because treatment for AIS includes self-care behaviors, such as wearing a brace and restricting activities, differences in health information acquired (regardless of quality) on the internet by adolescents and parents may result in different understandings, concerns, and expectations regarding the condition and treatments. Ultimately, these differences could result in stress and conflicts in the healthcare setting and at home regarding medical treatment decisions and adherence to treatment recommendations. Therefore, the current study assessed the type of information exchanged by adolescents and parents affected by AIS that are participating in OSGs, including whether the information exchanged was similar or different.

## Methods

The OSGs that were explored in this study were on the non-profit National Scoliosis Foundation (NSF) website where there are more than 8,000 members participating in many different AIS-related forums. This study was part of a larger research project assessing adolescent uncertainty surrounding AIS treatment (observation versus brace treatment), therefore two adolescent NSF forums, “Watching and Waiting” and “Bracing” were analyzed. However, despite the fact that the forums targeted adolescents undergoing observation or brace treatment, many participants were active in both forums simultaneously, discussed both observation and brace treatment, and many were parents of a child with AIS. Therefore, the authors analyzed the type of information exchanged by adolescents compared to parents instead of observation compared to brace treatment. Only posts that were available to the public were analyzed. The first author’s institutional review board (IRB) determined this study to be non-human subjects research.

### Participants

Preliminary analysis of more than 8,000 posts was conducted to identify gender, age group (adolescent or parent of a child with AIS), stage of illness/treatment, and topics relevant to the participants. After reviewing all the posts in the two NSF forums, 605 individuals were identified as being either an adolescent with AIS (*n* = 305) or a parent of a child with AIS (*n* = 300). All posts from these two populations were downloaded and each participant’s posts were numbered. One post from each participant was randomly selected using a random number generator. The dates of these posts ranged from November 2003 through to August 2014.

### Instrumentation

Categories and subcategories were coded based on the preliminary analysis and establishment of inter-rater reliability. Each information exchange was coded as either providing information or seeking information. Research indicates that the amount and type of illness-related information that an individual seeks is influenced by stage of illness, treatment experience, and consequences of the illness and treatment [12]. Therefore, if mentioned in the post, participants were coded as being recently diagnosed and having undergone (consequences) or currently undergoing common AIS stages of treatment progression (observation, brace, surgery) [13].

The type of information exchanged was coded into three major categories (AIS-specific, brace-specific, and other biomedical information). The AIS-specific subcategories included the following: causes/diagnosis/progression, co-morbid conditions/pain, functioning, and appearance/deformity. Brace-specific information included the following subcategories: how many hours per day of brace wear (recommended or actual), how long in months or years having undergone or undergoing brace treatment, types of braces, brace non-adherence (mentions of not wearing brace per recommendation), brace effectiveness, physical appearance, brace comfort, daily functioning, exercise/sports, and clothes/dressing. Finally other biomedical information subcategories captured information regarding: doctors/hospitals, and research/resources.

The first author and a graduate student read through randomly selected posts from the two OSGs and recorded the presence of information exchanged regarding the categories and subcategories. The two coders met to discuss discrepancies, review decision rules, and combine variable subcategories (if necessary), until acceptable reliabilities were achieved. Acceptable reliability was based on Krippendorff’s alpha scores that were greater than 0.80 [14]. After reliabilities were achieved, the two coders independently analyzed 15 % (*n* = 90) of the sample. The inter-coder reliability coefficients for the major information subcategories ranged from 0.87 to 0.97.

### Statistical analysis

The coding instrument data collection tool was developed using the REDCap data collection software. After the randomly selected posts had been coded, the data was exported and the statistical analyses were conducted using SPSS 21 and SAS Enterprise Guide 4.3. Pearson’s Chi-Square was conducted for comparisons between and within the adolescent and parent participants.

## Results

Table 1 describes adolescent and parent characteristics, including stage of illness/treatment, and types of AIS-related, brace-related, and other biomedical information exchanged. As far as stage of illness/treatment there were significantly more parents (*p* < 0.001) participating in the OSGs that had a child that was recently diagnosed with scoliosis compared to adolescents that were recently diagnosed. There were significantly more adolescents participating in the OSGs that had undergone or were currently undergoing brace treatment (*p* = 0.004) compared to parents of a child in this group. Compared to adolescents, parents were exchanging significantly more information regarding doctors/hospitals (*p* = < 0.001).

**Table 1:** Characteristics of Adolescents with AIS and Parents of a Child with AIS in OSGs

	Adolescents (*n* = 305)	Parents (*n* = 300)	*p*
Gender			.770
Female	205 (89 %)	208 (92 %)	
Male	25 (11 %)	18 (8 %)	
Current/past treatments			
Just diagnosed	28 (9 %)	90 (20 %)	< .001
Observation	23 (8 %)	27 (9 %)	.515
Brace treatment	196 (64 %)	158 (53 %)	.004
Scheduled for surgery	14 (5 %)	17 (6 %)	.549
Have had surgery	31 (10 %)	21 (7 %)	.165
Type of information exchange			.180
Providing information	160 (52 %)	141 (47 %)	
Seeking information	145 (48 %)	159 (53 %)	
AIS-specific information			
Causes, progression and/or diagnosis	96 (31 %)	145 (48 %)	< .001
Co-occurring conditions and/or pain	45 (15 %)	42 (14 %)	.792
Physical functioning	25 (8 %)	20 (20 %)	.474
Appearance	38 (12 %)	16 (5 %)	.002
Brace-specific information			
Daily/nightly brace wear	40 (13 %)	35 (12 %)	.589
Monthly/yearly brace wear	35 (11 %)	24 (8 %)	.150
Types of brace	62 (20 %)	75 (25 %)	.170
Non-adherence to brace wear	20 (7 %)	13 (4 %)	.230
Effectiveness of brace treatment	52 (17 %)	63 (21 %)	.216
Physical appearance in front of others	58 (19 %)	26 (9 %)	< .001
Comfortableness of wearing the brace	57 (19 %)	47 (16 %)	.325
Daily physical functioning in brace	31 (8 %)	27 (9 %)	.725
Participation in sports/exercise	7 (2 %)	17 (6 %)	.034
What to wear/clothes and/or how to dress	33 (11 %)	27 (9 %)	.455
Other biomedical information			
Doctors/hospitals	14 (5 %)	63 (21 %)	< .001
Research/resources	11 (4 %)	16 (5 %)	.305

*Note*. *p*-value is two-sided, significance at 0.05

Table 2 displays most to least frequent of the four AIS-specific and the ten brace-specific information subcategories. Despite information about AIS-related causes, progression, and/or diagnosis being the most frequent information exchange for both adolescents (*n* = 96) and parents (*n* = 145), there were significantly more parents (*p* < 0.001) exchanging (providing and seeking) this information compared to adolescents. Brace-related concerns about appearance was the third most frequent information exchange by adolescents (*n* = 58), which was significantly more (*p* < 0.001) than ninth ranking for parents (*n* = 26). Concerns about AIS-specific deformities and appearance ranked eighth for adolescents (*n* = 38) and was mentioned significantly more (*p* = 0.002) compared to the thirteenth ranked subcategory for parents of a child with AIS (*n* = 13). Finally, while information regarding brace-specific concerns about participation in sports ranked low in frequency in both age groups, parents (*n* = 17) had moderately significantly more mentions (*p* = 0.034) compared to adolescents (*n* = 7).

**Table 2:**
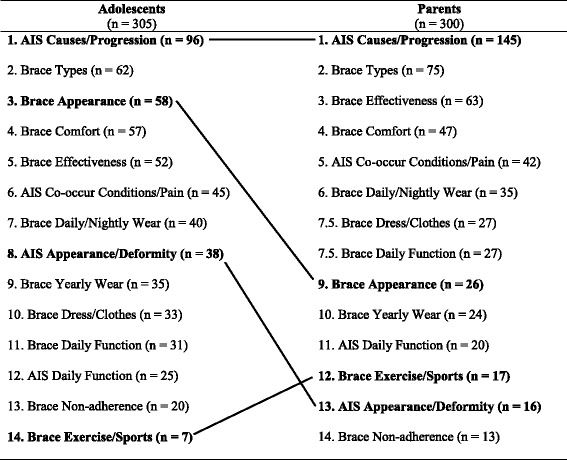
Adolescents’ and Parents’ AIS-specific and Brace-Specific Information Exchanges by Most to Least Frequently Mentioned Subcategories

*Note*. *p*-value is two-sided, significance at 0.05

When comparing message exchanges that were providing information to messages that were seeking information, there were several significant differences within each age group based on stage of diagnosis/treatment, and based on the three information categories (AIS-related, brace-related, and other biomedical). Table 3 summarizes the frequencies and differences in the adolescent and parent groups based on whether the individual was providing information or seeking information. Adolescents were seeking significantly more information than providing it when they were just diagnosed (*p* = 0.021) and were undergoing observation (*p* = 0.002). Parents of a child that had been recently diagnosed with AIS were also seeking information significantly more than they were providing information (*p* < 0.001). Adolescents that experienced brace treatment were providing significantly more information compared to seeking (*p* = 0.008). Both adolescents and parents of a child were providing information significantly more than they were seeking information when they were scheduled to have surgery (*p* = 0.011 and *p* = 0.012, respectively) and when they had already had surgery (both groups, *p* < 0.001).

**Table 3:** Characteristics of Adolescents and Parents Information Exchanges (Seeking or Providing) in OSGs

	Adolescents			Parents		
	Providing information (*n* = 160)	Seeking information (*n* = 145)	*p*	Providing information (*n* = 141)	Seeking information (*n* = 159)	*p*
Past/current treatment						
Just diagnosed	9	19	.021	8	53	< .001
Observation	5	18	.002	10	17	.278
Brace	114	82	.008	82	76	.073
Having surgery	12	2	.011	13	4	.012
Had surgery	28	3	< .001	19	2	< .001
AIS-specific						
Causes	41	55	.021	59	86	.034
Co-occurring	16	29	.014	9	33	< .001
Functioning	7	18	.011	9	11	.085
Appearance	10	28	.001	12	4	.070
Brace-specific						
Daily/nightly	27	13	.041	19	16	.359
Monthly/yearly	24	11	.043	14	10	.247
Types of	39	23	.065	40	35	.205
Non-adherence	12	8	.486	5	8	.529
Effectiveness	39	13	< .001	32	31	.498
Appearance	36	22	.104	21	5	< .001
Comfort	29	28	.791	28	19	.060
Functioning	16	15	.921	11	16	.495
Exercise/sports	4	3	.802	7	10	.621
Clothes/dress	20	13	.322	16	11	.182
Other biomedical						
Doctors/hospitals	7	7	.851	23	40	.061
Research/resources	9	2	.047	13	3	.005

*Note*. *p*-value is two-sided, significance at 0.05

Adolescents were seeking significantly more than providing AIS-specific information regarding causes/progression/diagnosis (*p* = 0.021), co-morbid conditions/pain (*p* = 0.014), daily physical functioning (*p* = 0.011), and appearance/deformity concerns (*p* < 0.001). Adolescents were providing significantly more than seeking brace-specific information regarding daily/nightly brace wear (*p* = 0.041), monthly/yearly brace wear (*p* = 0.043), and effectiveness of brace treatment (*p* < 0.001). Parents were seeking information significantly more compared to providing regarding AIS causes/progression/diagnosis (*p* = 0.034) and co-morbid conditions/pain (*p* < 0.001). Finally, parents were providing information significantly more than seeking information concerning appearance in the brace (*p* < 0.001).

## Discussion

Results from this study indicate that there are similarities regarding the reasons adolescents with AIS and parents of a child with AIS are participating in the AIS-related OSGs. The most sought after information by both adolescents and parents was regarding the causes, progression and/or diagnosis of AIS. Two of the top four most frequent types of information exchanged for both adolescents and parents were regarding types of brace and brace comfort. These findings suggest that, regardless of stage of diagnosis/treatment, adolescents and parents might not be receiving enough information from healthcare providers and are therefore going to the OSGs to exchange (provide and seek) information about other’s experiences with the causes, progression and/or diagnosis of AIS and about other’s experiences with particular types of braces, in particular the comfort of different braces.

Results from this study also found differences between adolescents and parents participating in the AIS-related OSGs regarding their stage of diagnosis/treatment and regarding the type of AIS-related and brace-related information being exchanged. In general, it appears that parents immediately go online as soon as a child is diagnosed with AIS to seek information regarding the causes of AIS and health conditions associated with AIS. While adolescents wait until they have experienced brace treatment to participate in OSGs for the purpose of seeking information regarding AIS-related causes/diagnosis/progression and for providing information regarding their experiences, in particular how much they wore the brace (per day and years) and whether brace treatment was effective. These findings suggest that, compared to the other, adolescents and parents of a child with AIS might need additional AIS-related information based on their stage of diagnosis and treatment. For example, compared to adolescents, at the onset of AIS diagnosis parents might benefit from additional information from healthcare providers regarding AIS-related information regarding causes/diagnosis/progression, and co-occurring conditions/pain. While adolescents that have undergone or are currently undergoing brace treatment might benefit from additional information from healthcare providers regarding any AIS-related information, in particular causes/diagnosis/progression.

Findings from this study also suggest that adolescents might benefit from additional support and coping strategies regarding other adolescent and parent experiences with AIS-related appearance/deformity concerns, brace wear appearance-related concerns, and regarding relationships between brace wear and brace effectiveness. According to Brashers et al. [13], this type of information and social support can assist in the following ways: facilitate skills development (i.e. giving tips regarding how they changed for PE class with the brace on); giving or receiving acceptance or validation (i.e. validating others frustration regarding brace wear); and encouraging perspective shifts (i.e. telling others that they too were frustrated but wearing the brace was just a short-time in one’s life).

In an attempt to provide families affected by AIS with additional information and support that they may be unable to address in the healthcare setting, some healthcare providers are referring families to AIS-related websites [15]. While the internet can be a helpful tool for empowering families dealing with AIS, current research indicates that the quality of scoliosis-specific information regarding AIS and treatments is poor, irrelevant, and misleading [16–18]. Findings from this study may provide insight into whether individuals participating in OSGs are negatively or positively biasing information compared to the experiences of individuals that are not participating in the OSGs and compared to AIS-related research findings. For example, adolescents that didn’t wear their brace and didn’t need surgery might be unintentionally providing support that encourages others to not wear their brace as recommended. On the flip side, adolescents that wore their brace as recommended but still needed to have surgery might be participating in the OSGs to vent their frustration, which may have encouraged others to not adhere to brace wear recommendations.

In summary, healthcare providers need to be aware of how families dealing with AIS are exchanging health information on the internet including: type of format (such as OSGs), who is participating (adolescents and parents), when they are participating (stage of diagnosis/treatment), and the type of and accuracy of information that is being provided. This type of knowledge may help the healthcare provider address any confusion that information from the internet may be generating, in particular information from other adolescents in OSGs regarding their brace-wear experience and whether it was effective. While highlighting the fact that everyone’s experience is different, healthcare providers can refer families dealing with AIS to additional quality internet resources for social support and for evidence-based information, such as recent results from the Bracing in Adolescent Idiopathic Scoliosis Trial (BrAIST).

BrAIST research findings indicate that bracing significantly decreased the progression of high-risk curves to the threshold of needing surgery (>50° largest Cobb angle) in adolescents with AIS [19]. In addition, BrAIST findings indicate that physical appearance and quality-of-life are not negatively impacted by nor does it negatively impact, brace wear compared to individuals undergoing observation or brace wear adherence [20, 21]. BrAIST results, along with the nature and complexity of the condition of AIS and its treatment, can be communicated to families dealing with AIS to help address some of the information, questions, and possibly misinformation that are being generated in AIS-related OSGs.

Because AIS is a less-common condition, for some adolescents and parents, the internet is the only place they may connect with other individuals who have similar experiences and obtain information regarding AIS that is relevant to their lives. Findings from this study highlight the potential for providing web-based support to address the types of information needs and support of families dealing with AIS. Furthermore, findings from this study suggest that the web-based support needs to include information that is tailored to adolescents and parents, to age group and to stages of diagnosis/illness, and provide accurate, evidenced-based information. Ultimately, this type of information and social supportive intervention may improve health communication within and outside of the healthcare setting.

## Conclusion

This study is the first to examine types of health information exchanged in OSGs by adolescents with AIS and parents of a child with AIS. Results from this study suggest that adolescents and parents are seeking and providing information regarding the condition of AIS, AIS-treatment, and other biomedical information in OSGs. This study found differences between adolescents and parents in the stages of diagnosis/treatment and in the type of information being exchanged in AIS-related OSGs, including whether individuals are providing or seeking certain types of information. Therefore, information that is being provided to adolescents with AIS and parents of a child with AIS should take into consideration the age of the individual and the stage of diagnosis/treatments. This study of adolescents’ and parents’ information exchange in AIS-related OSGs may help inform priorities in health communication among healthcare providers and adolescents with AIS and parents of a child with AIS, and between adolescents and parents that are dealing with AIS.

## Abbreviations

AIS, adolescent idiopathic scoliosis; IRB, institutional review board; NSF, National Scoliosis Foundation; OSG, online support groups
